# A System of Practical Medicine

**Published:** 1885-12

**Authors:** 


A SYSTEM OF PRACTICAL MEDICINE. By American Authors. Edited by William Pepper, M. D. LL. D., Prevost and Professor of the Theory and Practice of Medicine and of Clinical Medicine in the University of Pennsylvania, assisted by Louis Starr, M. D., Clinical Professor of Diseases of Children in the Hospital of the University of Pennsylvania. Vol. III, Diseases of the Respiratory, Circulatory Hæmatopoietric systems. Philadelphia: Lea Bros. & Co. 1885.

The favorable reception of the first two volumes of this work by the medical public throughout this country affords a reasonable presumption that this third well-stored volume will be equally appreciated, and it is in some respects entitled to greater consideration, and to be held in higher estimation by practitioners, than its predecessors.

The elaborate article on diseases of the blood and blood-glandular system occupies sixty-eight pages, being the largest contribution to the volume. Another on diseases of the substance of the heart, by the same author, has thirty-seven pages. That on pulmonary phthisis fills fifty-five pages; that on endocarditis and cardiac valvular diseases takes up forty-seven pages, and one on croupous pneumonia, by the same author, has only one page less, containing forty-six pages. The paper on the diseases of the pericardium contains thirty-four pages, and none of the others exceed thirty pages, while there are some of five or six pages.

An able article of twenty pages by the editor lays great stress upon diagnosticating catarrhal pneumonia. In all, from the longest to the shortest, there are evidences of much research and great painstaking in the preparation of the articles, and these several treatises present the most complete and thorough consideration of the various subjects included in this volume that can be found in medical literature.

The alphabetical index at the close of the volume facilitates greatly the search for information upon any special topic, and enhances it, as a book of reference, to the student or the medical practitioner.

It is due the publishers to state that the mechanical execution and typographical finish of the work are simply unexceptionable in their high order of excellence, and we congratulate the medical reader upon such an acquisition to his library as this volume of 1,032 pages adds to the series composing the system of medicine.

